# Supplementary material for: Targeting Oncogenic Mutant p53 and BCL-2 for Small Cell Lung Cancer Treatment
Source: Int J Mol Sci. 2023 Aug 23;24(17):13082. doi: 10.3390/ijms241713082 (PMC10487506; doi:10.3390/ijms241713082)
Supplement: Supplementary file 1 [file ijms-24-13082-s001.zip › ijms-2570234-supplementary.pdf]

Figure S1

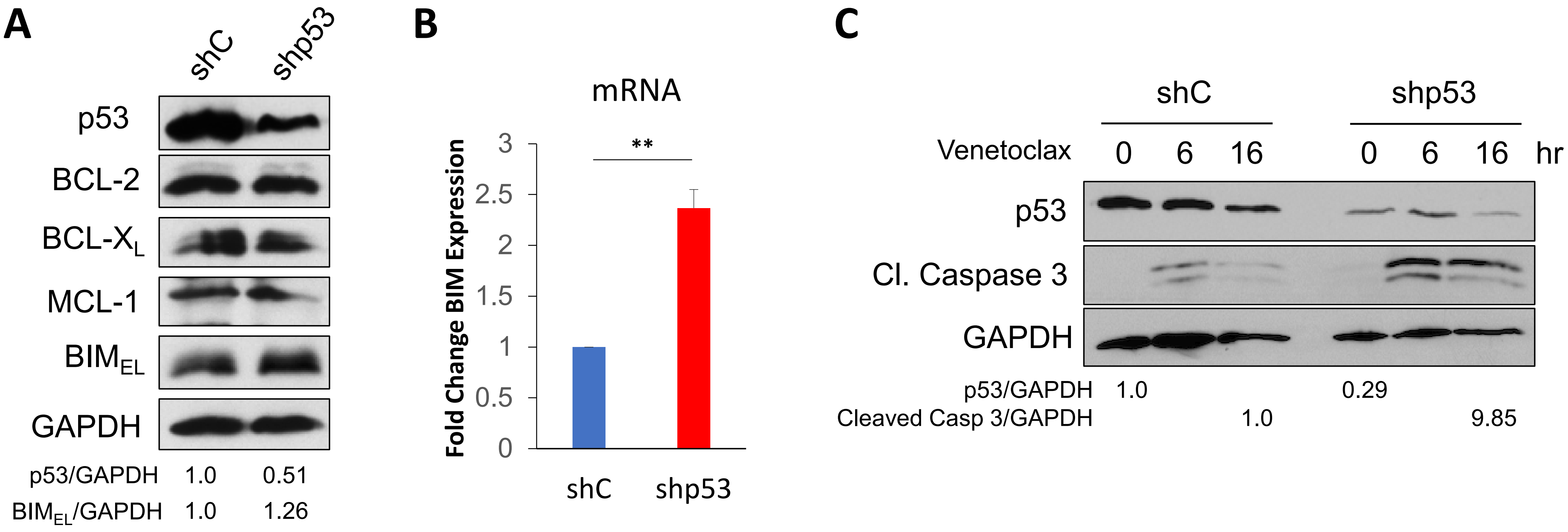

Figure S1. Down-regulation of Onc-p53 induces BIM and sensitizes SCLC to venetoclax in H211 cells. (A) Onc-p53 was downregulated by shRNA (shp53) in H211 cells. The levels of BCL-2 family proteins compared to scrambled shRNA control (shC) were determined by Western blots with the indicated antibodies. (B) The levels of *Bim* mRNA in H211 shp53 and scrambled RNA control cells were determined by qRT-PCR. (**C**) H211 shp53 and scrambled RNA control cells were treated with 1 μM venetoclax. Total cell extracts were prepared at the indicated time points and were subjected to Western blots with the indicated antibodies. **p<0.01. Error bars equal SD.

Figure S2

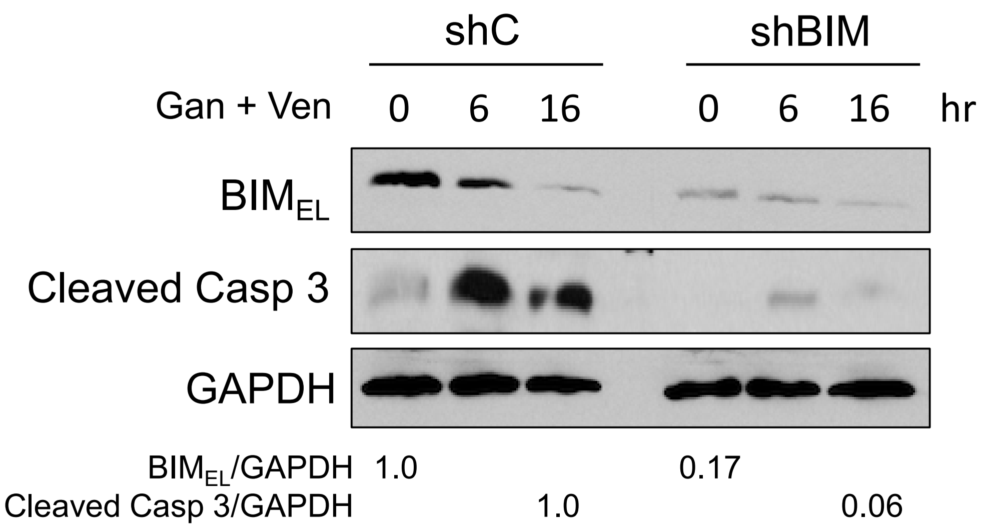

Figure S2. Down-regulation of BIM mitigates cell death induced by venetoclax and ganetespib in H211 cells. H211 shBIM and scrambled RNA control cells were treated with 1 μM venetoclax and/or 50 nM ganetespib. Total cell extracts were prepared at the indicated time points and were subjected to Western blots with the indicated antibodies.

Figure S3

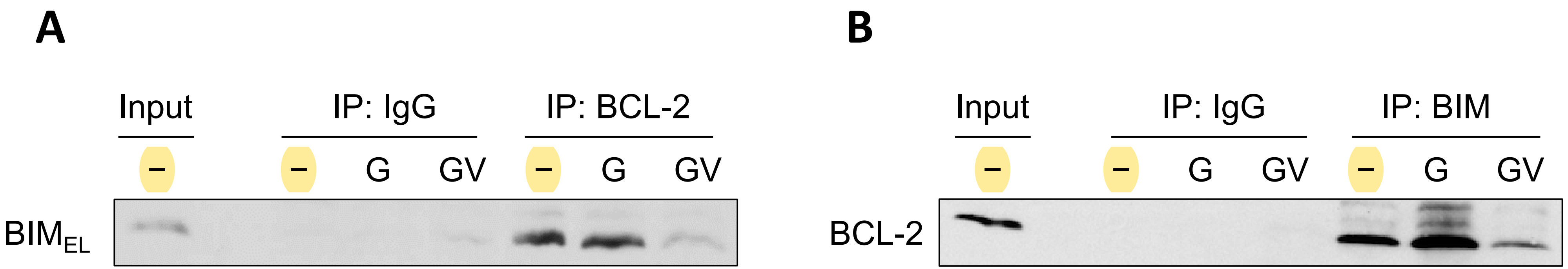

Figure S3. Co-immunoprecipitation with normal IgG indicates that anti-BCL-2 and anti-BIM antibodies are specific. H1048 cells were treated with 1 μM venetoclax and/or 50 nM ganetespib for 8 hours. Total cell lysates were subjected to immunoprecipitation with (A) normal mouse IgG and anti-BCL-2 antibodies, (B) normal rabbit IgG and anti-BIM antibodies. Western blot analyses were carried out on precipitated samples with the indicated antibodies. Input demonstrated the integrity of each cell lysate.

Original Western Blots

In 300 dpi

Fig 1A

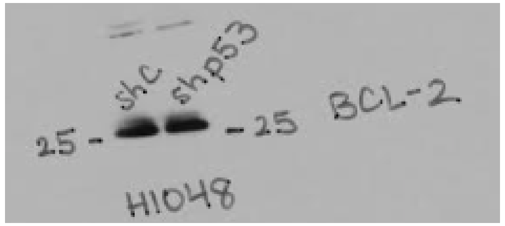

BCL-2 (25 kD)

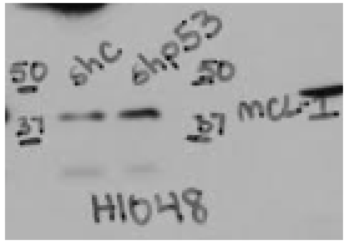

MCL-1 (40 kD)

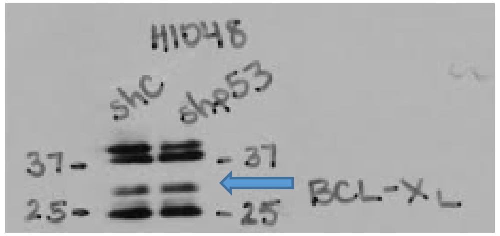

BCL-XL (30 kD)

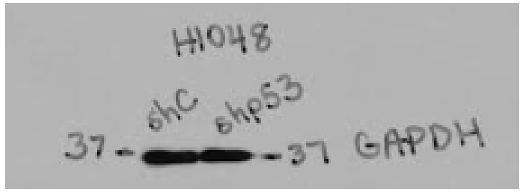

GAPDH (37 kD)

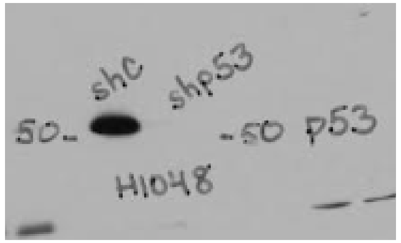

p53 (53 kD)

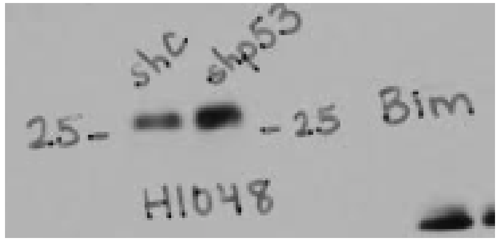

BIM_{EL} (25 kD)

Fig 1C

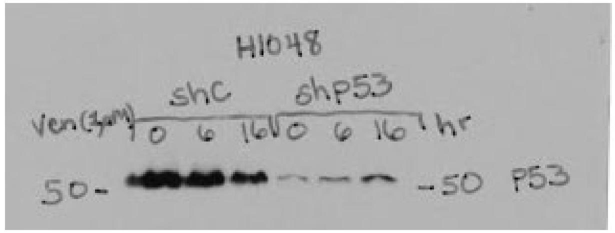

p53 (53 kD)

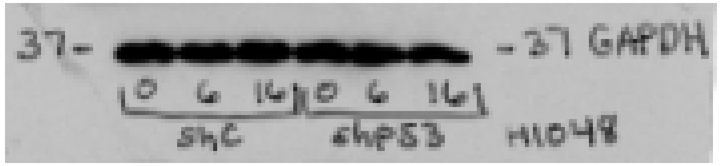

GAPDH (37 kD)

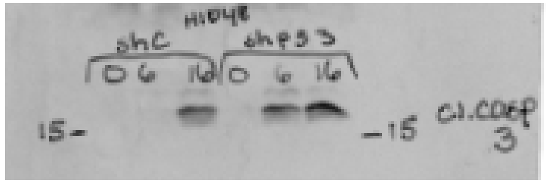

Cleaved Caspase 3 (19 kD)

Fig 2A

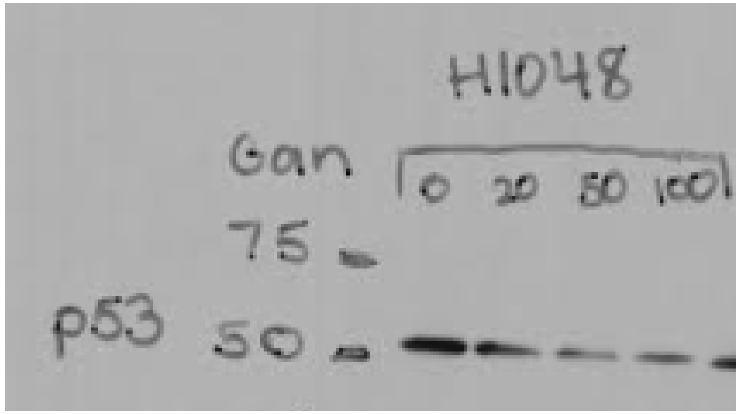

p53 (53 kD)

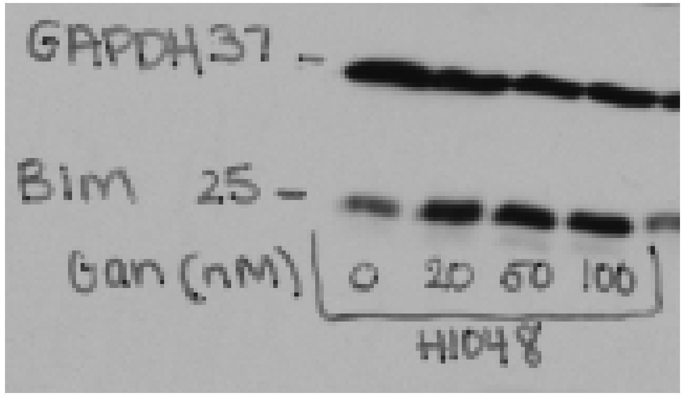

GAPDH (37 kD) and BIM_{EL} (25 kD)

Fig 2D

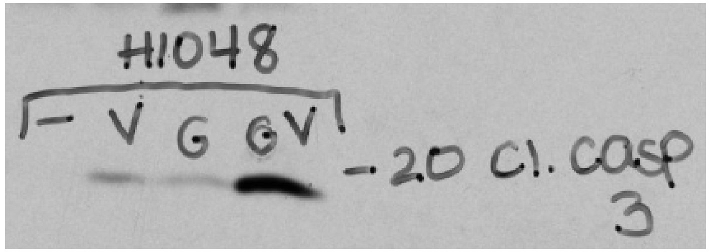

Cleaved Caspase 3 (19 kD)

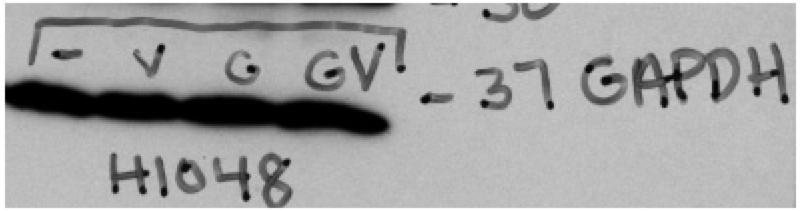

GAPDH (37 kD)

Fig 4B

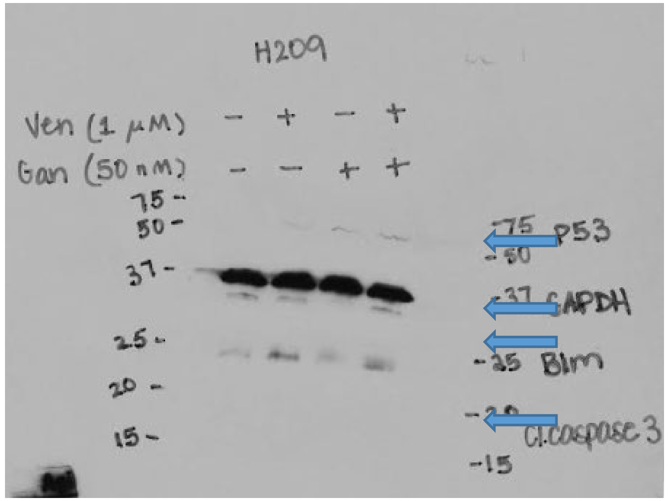

p53 (53 kD), GAPDH (37 kD), BIM_{EL} (25 kD), Cleaved Caspase 3 (19 kD)

Fig 5B

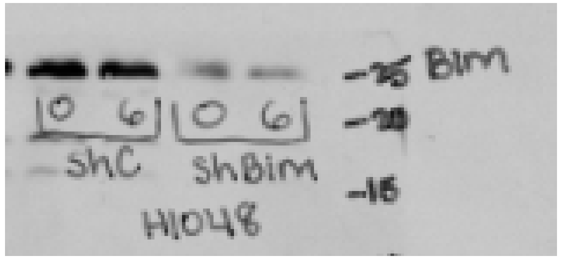

BIM_{EL} (25 kD)

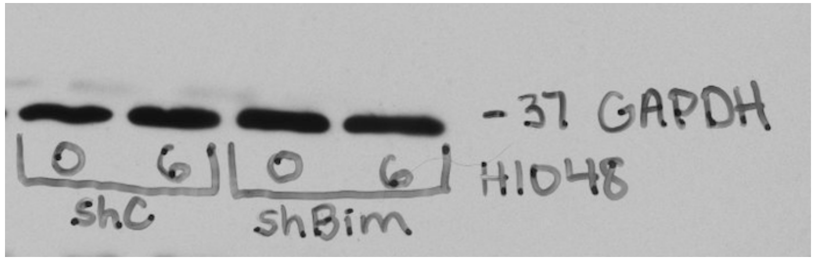

GAPDH (37 kD)

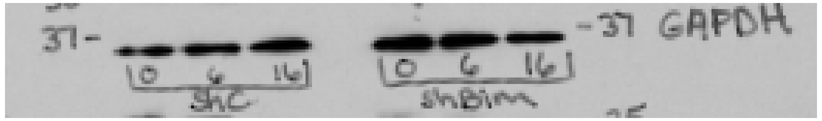

GAPDH (37 kD)

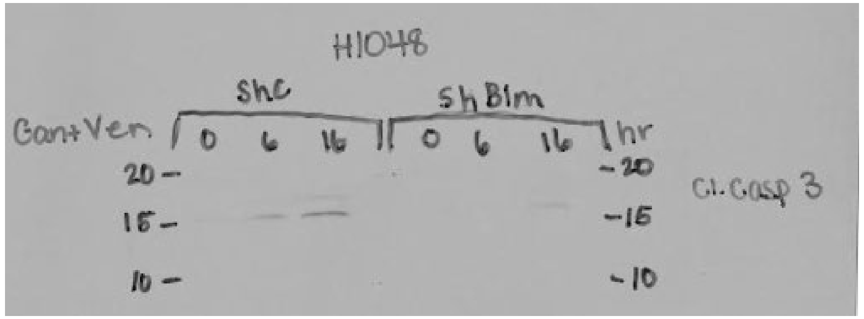

Cleaved Caspase 3 (19 kD)

Fig 6A

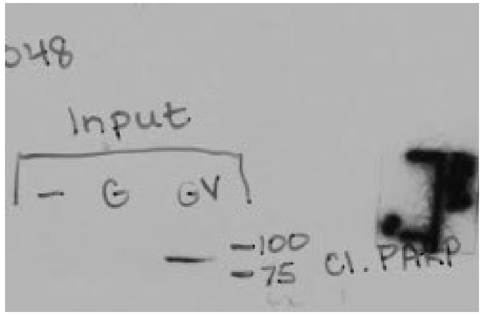

Cleaved PARP (89 kD)

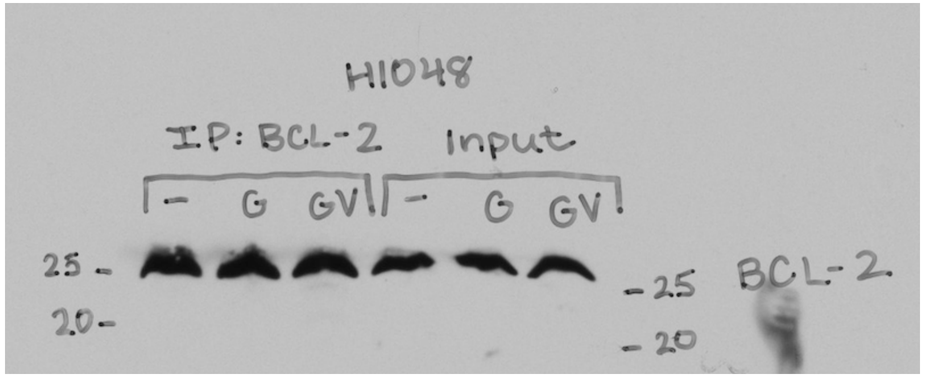

BCL-2 (25 kD)

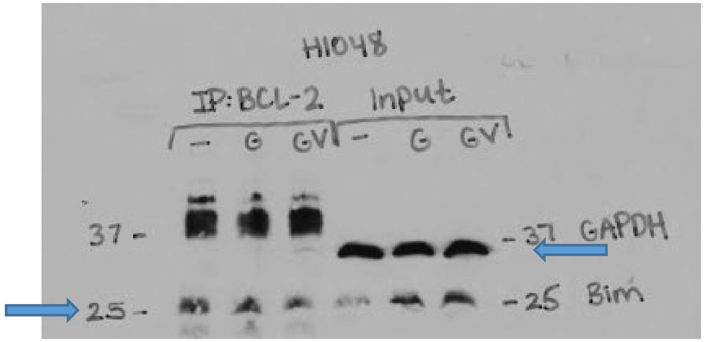

GAPDH (37 kD) and BIM$_{EL}$ (25 kD)

Fig 6B

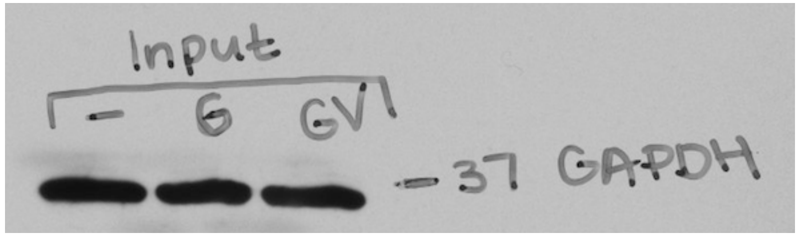

GAPDH (37 kD)

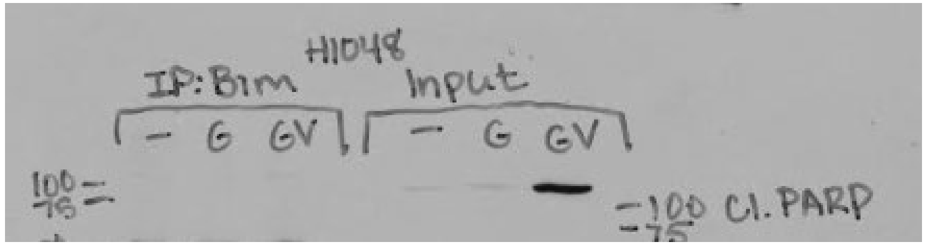

Cleaved PARP (89 kD)

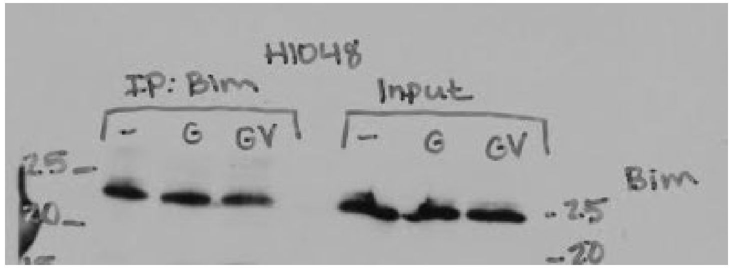

BIM$_{EL}$ (25 kD)

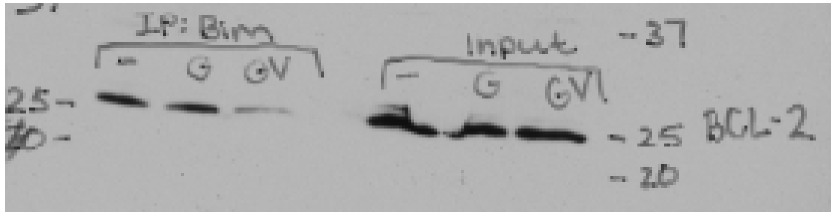

BCL-2 (25 kD)

Fig 6C

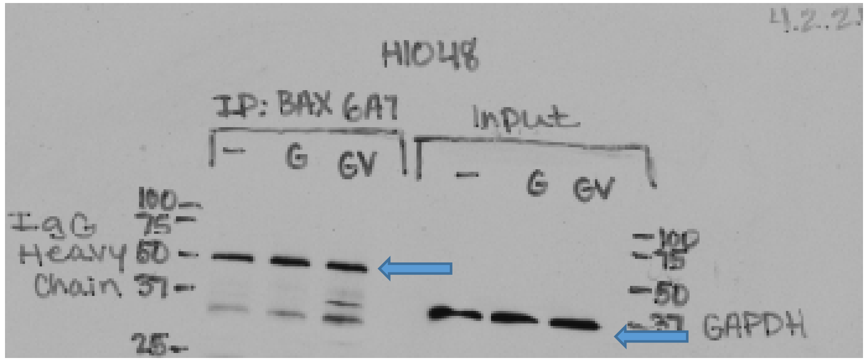

IgG heavy chain (~50 kD) and GAPDH (37 kD)

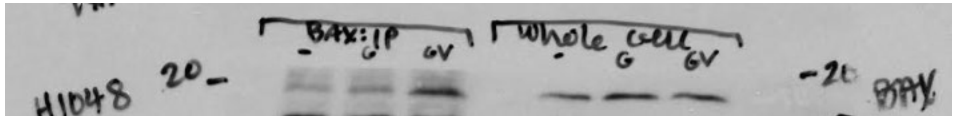

BAX (20 kD)

Fig S1A

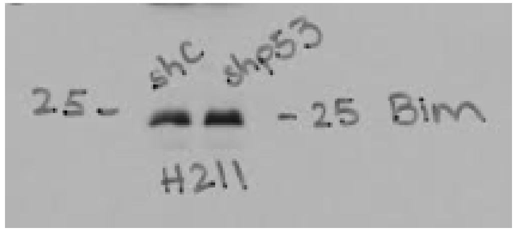

BIM$_{EL}$ (25 kD)

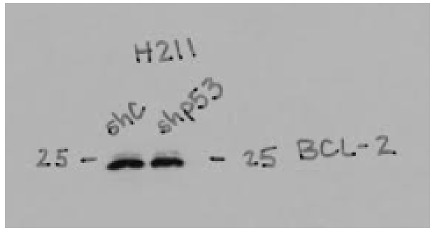

BCL-2 (25 kD)

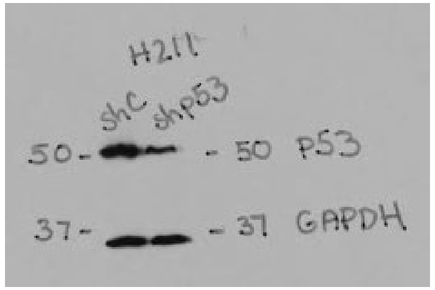

p53 (53 kD) and GAPDH (37 kD)

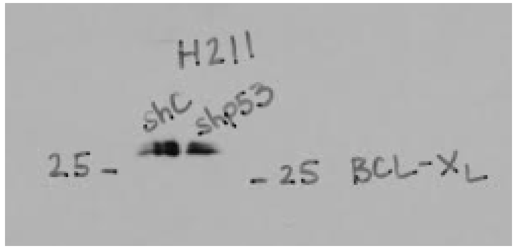

BCL-XL (30 kD)

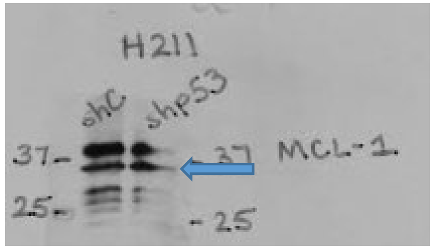

MCL-1 (40 kD)

Fig S1C

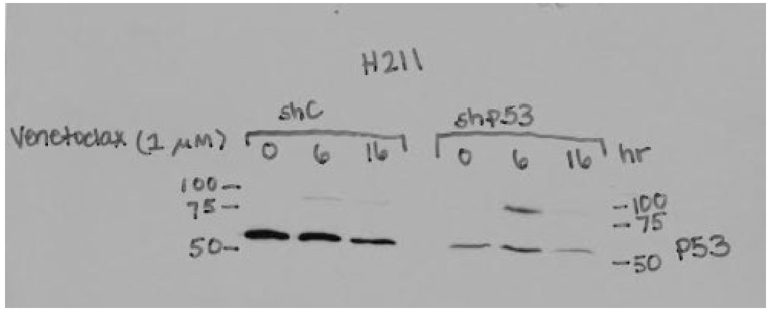

p53 (53 kD)

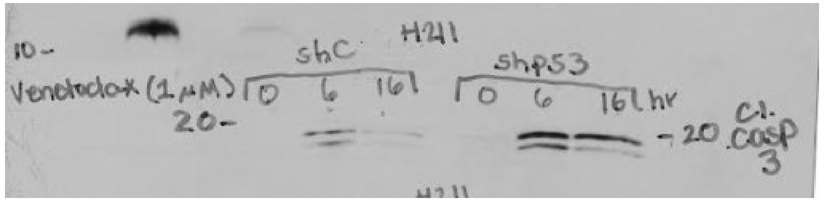

Cleaved Caspase 3 (19 kD)

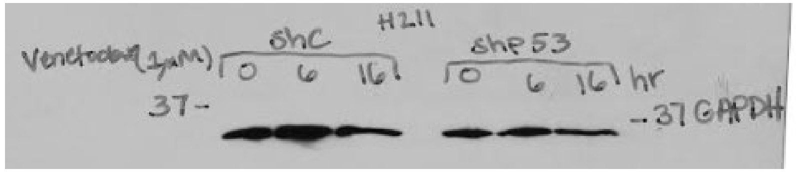

GAPDH (37 kD)

Fig S2

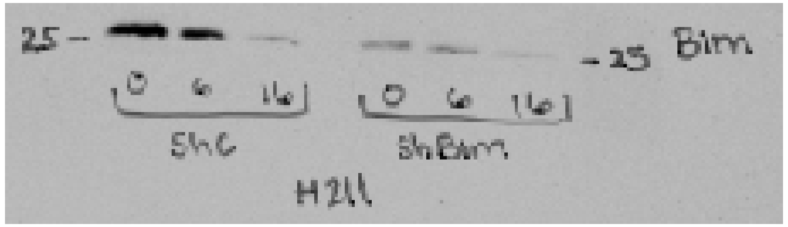

BIM_{EL} (25 kD)

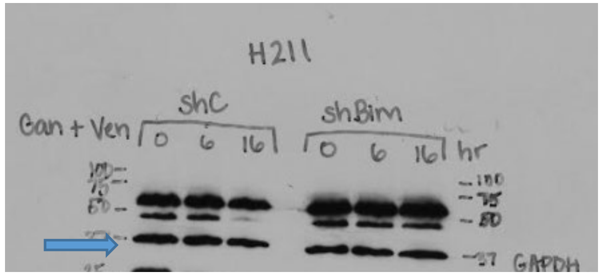

GAPDH (37 kD)

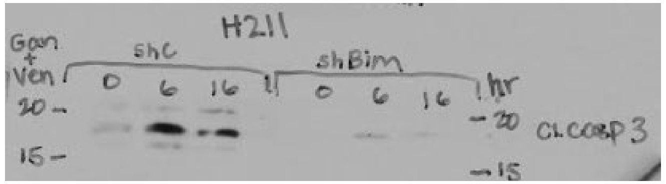

Cleaved Caspase 3 (19 kD)

Fig S3

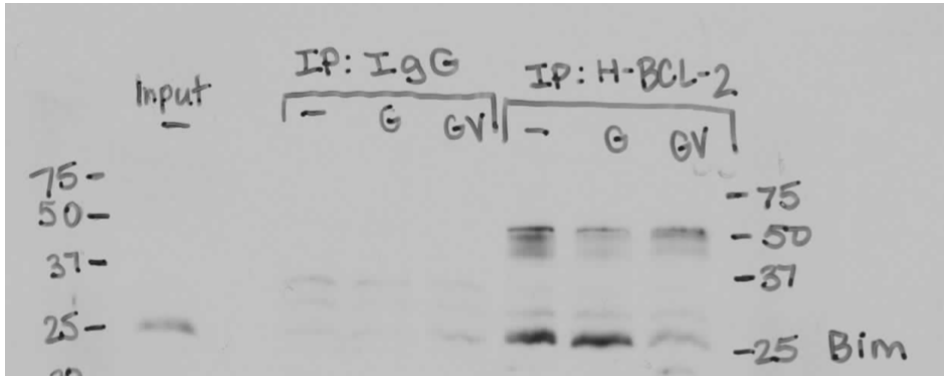

BIM$_{EL}$ (25 kD)

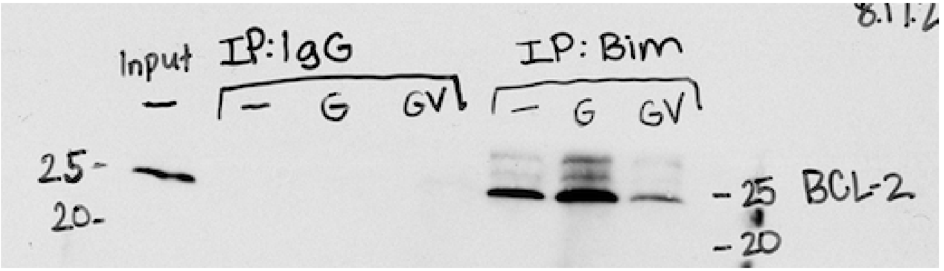

BCL-2 (25 kD)
